# Assessing parameter uncertainty in small-n pharmacometric analyses: value of the log-likelihood profiling-based sampling importance resampling (LLP-SIR) technique

**DOI:** 10.1007/s10928-020-09682-4

**Published:** 2020-04-04

**Authors:** Astrid Broeker, Sebastian G. Wicha

**Affiliations:** Department of Clinical Pharmacy, Institute of Pharmacy, University of Hamburg, Bundesstraße 45, 20146 Hamburg, Germany

**Keywords:** Parameter uncertainty, Small datasets, Sampling importance resampling, Bootstrap, Log-likelihood profiling, LLP-SIR

## Abstract

Assessing parameter uncertainty is a crucial step in pharmacometric workflows. Small datasets with ten or fewer subjects appear regularly in drug development and therapeutic use, but it is unclear which method to assess parameter uncertainty is preferable in such situations. The aim of this study was to (i) systematically evaluate the performance of standard error (SE), bootstrap (BS), log-likelihood profiling (LLP), Bayesian approaches (BAY) and sampling importance resampling (SIR) to assess parameter uncertainty in small datasets and (ii) to evaluate methods to provide proposal distributions for the SIR. A simulation study was conducted and the 0–95% confidence interval (CI) and coverage for each parameter was evaluated and compared to reference CIs derived by stochastic simulation and estimation (SSE). A newly proposed LLP-SIR, combining the proposal distribution provided by LLP with SIR, was included in addition to conventional SE-SIR and BS-SIR. Additionally, the methods were applied to a clinical dataset. The determined CIs differed substantially across the methods. The CIs of SE, BS, LLP and BAY were not in line with the reference in datasets with ≤ 10 subjects. The best alignment was found for the LLP-SIR, which also provided the best coverage results among the SIR methods. The best overall results regarding the coverage were provided by LLP and BAY across all parameters and dataset sizes. To conclude, the popular SE and BS methods are not suitable to derive parameter uncertainty in small datasets containing ≤ 10 subjects, while best performances were observed with LLP, BAY and LLP-SIR.

**Electronic supplementary material:**

The online version of this article (10.1007/s10928-020-09682-4) contains supplementary material, which is available to authorized users.

## Introduction

Nonlinear mixed-effects modelling has gained an important role in quantitative clinical pharmacology, drug development and therapeutic drug use [1, 2]. In many applications when non-linear mixed effects modelling is employed, small datasets containing ten or even fewer subjects appear regularly [3–5], for example due to ethical concerns, high risks, high costs, low availability of patient (sub)groups of interest to the study or multiple factors. Conclusions drawn from such ‘small-n’ studies depend on the estimated parameters as well as parameter uncertainty. Wrongly determined parameter uncertainty can lead to inaccurate stochastic simulation studies and might also adversely influence power calculations derived from clinical trial simulations [6, 7].

The calculation of the standard error (SE) based on the inverse of the Fisher information matrix of the covariance step is the fastest, easiest and most popular way to obtain an estimate of the parameter uncertainty [8]. However, parameter uncertainty via SE has been discussed critically: Numerical difficulties in the covariance step leading to implausible SE’s and the assumption of normal distribution of the SE-based confidence intervals (CI) are significant disadvantages of the SE technique [9, 10].

Bootstrapping (BS) is a well-accepted strategy for assessing parameter uncertainty. BS does not depend on a successful covariance step and allows for asymmetry of the derived CIs. The BS became a gold standard technique to evaluate parameter uncertainty and computation time is often seen as the only major drawback of the BS. However, heterogeneous sampling and unbalanced study designs violate the assumption that each sample contributes evenly balanced information leading to incorrect confidence intervals in these situations [11]. Accordingly, datasets with low subject numbers or low homogeneity of the information content between subjects might not be suitable for the BS technique [12].

Log-likelihood profiling (LLP, also referred to as objective function mapping) is another, less frequently used method to assess parameter uncertainty employing the likelihood ratio test [8]. As with BS, LLP also does allow for asymmetric confidence intervals. Its computational demand is usually between BS and SE. The main drawback of LLP is its univariate character, which hampers using the results of an LLP run for stochastic simulations, which preferably consider possible correlations between parameters. First steps towards an N-dimensional LLP were taken by Denney and colleagues in 2012, but this approach is not yet available to the pharmacometric community [13].

Bayesian methods (BAY) provide a posterior distribution of the parameters, which allows deriving 95% CI’s to assess parameter uncertainty [14]. They are faster as compared to BS and provide dense information within one run giving the posterior distribution, including correlation between parameters and possible asymmetry of confidence intervals. Thus, these approaches directly provide access to parameter uncertainty. In addition, BAY methods can be used sequentially after a frequentist estimation using the final parameter estimates as uninformative prior to derive confidence interval using the posterior distribution [14].

The sampling importance resampling (SIR) procedure was proposed to overcome some of the aforementioned limitations associated with the use of SE, BS and LLP [9]. SIR is faster than BS. It can be conveniently used through integration of the SIR algorithm into PsN [15, 16], and also computes asymmetric confidence intervals. SIR requires a proposal distribution to initiate the sampling procedure and commonly the SE, BS or an educated guess is utilized, but it is unclear which proposal distribution might provide optimal results, in particular in small datasets.

The aim of this study was to (i) systematically evaluate commonly used methods to assess parameter uncertainty in very small datasets containing 5 subjects, small datasets containing 10 subjects and regular datasets containing 50 subjects and (ii) to compare and define optimal methods to derive proposal distributions for SIR for application in small datasets.

## Methods

### Dataset and model

In order to evaluate the approaches to assess parameter uncertainty in small datasets, clinical trials with very small datasets, small datasets and regular datasets containing 5, 10 and 50 subjects, respectively, were simulated. The pharmacokinetic model used for the simulations was a common two-compartment model (Clearance (CL): 10 L/h, central volume of distribution (V1): 20 L, inter-compartmental Clearance (Q): 5 L/h, peripheral volume of distribution (V2): 30 L) with inter-individual variability on Clearance (IIVCL: 0.1 (variance, log-normal distribution) and the central volume of distribution (IIVV1: 0.1 (variance, log-normal distribution)) and a proportional residual error model (15% CV). No covariates or inter-occasion variabilities were included. The design of the simulation datasets was motivated by realistic examples, containing a rather high number of samples per patient (n = 10) measured in the first and third dosing occasions. The sampling times were optimized iteratively in simulation and estimation studies (data not shown) and set to 0.7, 1.2, 2, 5, 7, 16.7, 17.2, 19, 20, 23 h resulting in an unbiased sampling design. The simulated dosing regimen was 1000 mg every 8 h with an infusion duration of 30 min. The model was encoded in NONMEM 7.4.3 (ICON, Gaithersburg, MD, USA) and the analytical solution of the two-compartment model (ADVAN3) was used. FOCE-I (first order conditional estimate with interaction) was used for parameter estimation. The simulations, estimations and methods to determine parameter uncertainty were controlled using R 3.6.1 calling NONMEM and PsN 4.7.0 [15].

### Determination of confidence intervals

The 0–95% CIs of stochastic simulation and estimation studies (SSEs, n = 1000) performed with PsN were used as a reference for parameter uncertainty.

Clinical trial simulations (n = 1000) were performed with NONMEM and the parameters were re-estimated for each dataset. The parameter uncertainty in every simulated study was assessed with SE, BS, LLP, BAY and the SIR variants SE-SIR, BS-SIR and LLP-SIR, using either SE, BS or LLP as input to the proposal distribution. Statistics on potentially terminated runs of each technique were collected.

Based on the SE derived from the variance–covariance matrix, the 0%, 20%, 40%, 60%, 80%, 90%, and 95% CIs around the final parameter estimates were calculated.

Subject based BS (n = 1000) was performed in R calling NONMEM for every simulated dataset and the 2.5th–97.5th, 5th–95th, 10th–90th, 20th–80th, 30th–70th, 40th–60th and 50th percentile were evaluated to determine the respective CIs.

LLP was performed using the PsN routine with an objective function increase criterion of 3.84, 2.706, 1.642 and 0.455 assuming the Χ^2^ distribution with one degree of freedom to determine the 95%, 90%, 80% and 50% CI, respectively, as well as the maximum likelihood estimate corresponding to the 0% CI.

BAY was performed using two approaches, i.e. Markov Chain Monte Carlo (MCMC) and no-u-turn sampling (NUTS) MCMC Bayesian analysis. NONMEM 7.4.3 was used according to standard settings provided with uninformative priors [17]. The final parameter estimates derived by FOCE-I were used as priors. For MCMC and for NUTS, 30,000 and 2000 iterations were used, respectively. The 2.5th–97.5th, 5th–95th, 10th–90th, 20th–80th, 30th–70th, 40th–60th and 50th percentile of the posterior distribution were evaluated to determine the respective CIs. In addition, in an exploratory analysis, to derive an SSE reference for the BAY estimation, which might diverge from the FOCE-based SSE reference, the 0–95% CI of the mean of the posterior distribution across 1000 simulations and estimations with the true parameters as uninformative priors was used to determine the reference parameter uncertainty (MCMC-SSE and NUTS-SSE).

For SIR based on the proposal distribution provided by the variance–covariance matrix, the PsN routine was used, which provides a 0%, 40%, 80%, 90%, and 95% CI for each parameter. The PsN standard settings for SIR were used as recommended by Dosne et al. [16], i.e. five iterations with sample vectors of M = 1000, 1000, 1000, 2000, 2000 and resample vectors of m = 200, 400, 500, 1000, 1000, respectively. In case of a failed covariance step, an educated guess (20% relative standard error) was provided.

For the BS-SIR, the BS results that were generated as described above, were used as a proposal distribution via the PsN routine.

The LLP-SIR, newly introduced in this work, combines the results of the LLP as a basis for the proposal distribution for the SIR. The 95% CI was transformed to a relative standard error (rse) assuming a normal distribution (± 1.96, α = 0.05) with the difference between 2.5th and 97.5th percentile (P_2.5_ and P_97.5_) in relation to the median (P_50_) of the LLP result:

$$rse=\frac{\frac{{P}_{97.5}-{P}_{2.5}}{2\times 1.96}}{{P}_{50}}\times 100$$

The rse calculated for each parameter served as an educated guess in the SIR PsN routine. Relative standard errors > 200% were set to 200%.

### Evaluation of the shape and distribution of the confidence intervals

The median of the lower and upper limit of the CIs determined by the methods SE, BS, LLP, BAY, SE-SIR, BS-SIR and LLP-SIR in 1000 simulations, were compared to the SSE results. Additionally, the 80% CIs around 2.5th and 97.5th percentile, reflecting the variability of the lower and upper value of the parameter uncertainty across the 1000 simulations, were calculated and compared.

### Evaluation of coverage

The 95% coverage was calculated as the fraction of the simulations, where the 95% CI included the true value. When the determination of the parameter uncertainty and consecutively of the 95% CI failed, for instance, when the covariance step did not complete, this simulation was excluded for the calculation of the coverage of this method.

### Clinical dataset example

All methods, SE, BS, LLP, BAY, SE-SIR, BS-SIR and LLP-SIR, were also applied to a clinical dataset example [5] and the 95% CI by the different methods were compared. The dataset originating from a hemodialysis study with 11 patients contained 180 blood and 109 dialysate samples thereby represented a typical example for a small-n study. The model was a two-compartment model with two parameters describing the clearance of hemodialysis and of hemodiafiltration, respectively, and a covariate relationship of bilirubin on CL as well as IIV on CL, V1, Q and CL_CVVHD_ (clearance of continuous veno-venous hemodialysis).

## Results

### Central tendency of confidence intervals

**Fig. 1:**
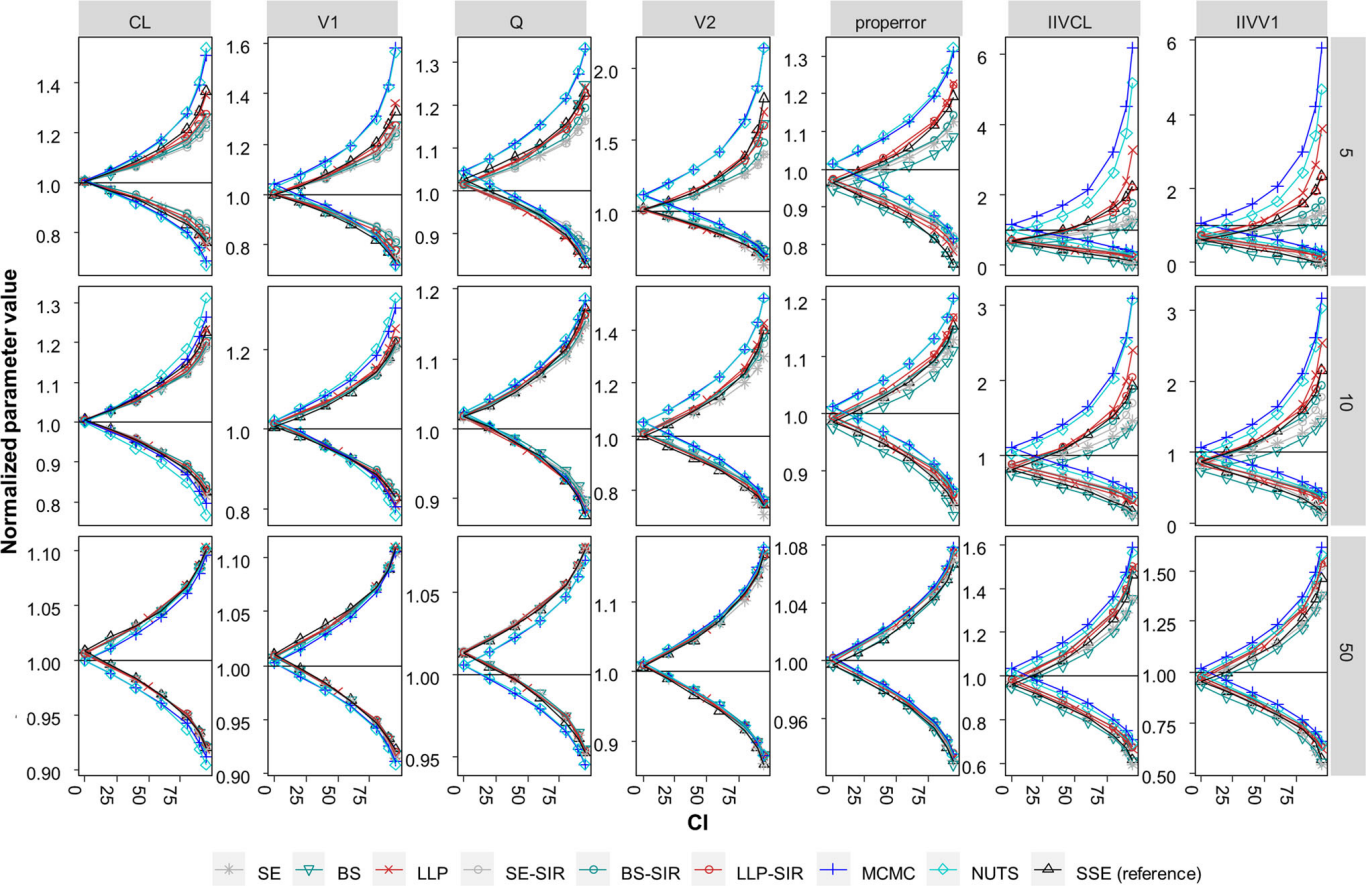
Normalized median of the parameter uncertainty expressed as 0–95% confidence intervals (CI) by parameter and evaluation approach across datasets containing 5–50 subjects compared to stochastic simulation and estimation (SSE) ‘reference’ CIs. CIs calculated from the SE: standard errors derived from the variance covariance matrix, BS: bootstrap, LLP: log likelihood profiling, SIR: sampling importance resampling, SE-SIR: SIR on SE based proposal distribution, BS-SIR: SIR on BS based proposal distribution, LLP-SIR: SIR on LLP based proposal distribution, MCMC: Markov Chain Monte Carlo Bayesian analysis, NUTS: no-u-turn sampling MCMC. IIV: interindividual variability. N = 1000 simulations

The central tendency of the CIs determined by the different methods is presented in Fig. 1. The median uncertainty (n = 1000 simulations) of each parameter was determined by SE, BS, LLP, BAY SE-SIR, BS-SIR and LLP-SIR and compared to SSE results. As expected, the 0–95% CIs were narrower with increasing study size. While CIs across methods diverged at small sample sizes, the CIs of the different approaches were more similar at higher sample sizes.

#### Structural parameters

For very small datasets containing five subjects, for the structural parameters, LLP provided the highest agreement with the SSE reference, whereas BAY overestimated the CIs and the other techniques tended to underestimate the CIs. For datasets ≥ 10 subjects, CIs derived for the structural parameters were less dependent on the chosen technique and closer to the SSE reference. For example, the boundaries of the 95% CI (normalized) for the dataset size of 10 subjects for CL ranged from 0.814 to 0.842 and from 1.19 to 1.23 for the lower and upper boundary, respectively, across the methods SE, BS, LLP and SIR. Larger CIs were provided by BAY (e.g. for CL the lower boundary ranged from 0.77 to 0.80 and upper boundary ranged from 1.27 to 1.31 in a dataset containing 10 subjects). As it can be expected, the SSE reference derived for the BAY methods was not always in line with the FOCEI-based SSE reference (Supplementary Fig. 1). However, the CIs by BAY in datasets containing ≤ 10 samples were also not always in line with the MCMC-SSE and NUTS-SSE, where CIs were increased as compared to the SSE reference (Supplementary Figure S1).

#### Variability parameters

Variability parameters, in particular CIs of IIV, were highly variable across methods. For example, the normalized 95% CI for the IIV of CL in a dataset of 10 subjects varied from 0.199–0.498 and 1.407–3.10, for the lower and upper boundary, respectively. A bias in the 0% CI, i.e. the point estimate, occurred for the datasets containing ≤ 10 subjects in the SSE reference and most pronounced in the BS, that by definition almost always underestimates variability in small datasets. The median derived by BAY methods overestimated the 0% CI. The bias was higher for the MCMC-SSE and NUTS-SSE (Supplement Figure S1). For the residual error, a similar, but less marked pattern as compared to the IIVs was observed.

The SE and BS in median underpredicted the reference CIs provided by the SSE of IIVCL and IIVV1. In contrast, the LLP overpredicted the upper boundary of the 90% and 95% CI of the IIV parameters. BAY was not in line with the SSE reference and overestimated the reference across all CIs for the lower and upper boundary. BAY was more in line with the MCMC-SSE and NUTS-SSE that differed substantially from the SSE reference for the IIV parameters in datasets containing ≤ 10 subjects. The lower boundary was overall captured better across all methods in datasets containing ≤ 10 subjects. In contrast to the upper boundary, SE and BS were more in line with the lower boundary of the SSE reference than LLP.

The result of the SIR was dependent on the proposal distribution: The BS-SIR provided too low 95% CIs for IIVs, similar to the SE based SIR. The LLP-SIR was best in line with the SSE reference CIs, even for very small datasets with only 5 subjects.

#### Termination statistics

The occurrences of terminations are presented in Table 1. The covariance step failed in 31 of 1000 estimations for datasets with 5 subjects and accordingly the SE-SIR was based on an educated guess (20% rse) in this case. Hence, in 969/1000 scenarios the variance covariance matrix was used as a proposal distribution in SE-SIR. Less than 2% of the BS runs terminated in 95% of simulations even for very small datasets with only 5 subjects. The statistics on the occurrence of relative standard errors > 200% by LLP being set to 200% for the LLP-SIR can be found in Supplementary Table 1.

**Table 1:** Statistics on the termination of parameter uncertainty determination by method

Method	Number of terminations (n = 1000 simulations)		
Subjects in dataset	5	10	50
SE	31	1	0
BS^a^ (mean, 5th, 95th percentile)	3.1 (0, 17)	0.09 (0, 1)	0.002 (0, 0)
LLP	0	0	0
SE-SIR^b^	0	0	0
BS-SIR	8	0	0
LLP-SIR	2	0	0
MCMC	0	0	0
NUTS	0	0	0

^a^Terminated runs per n = 1000 BS over 1000 simulations

^b^Proposal distribution was SE in 969 simulations and educated guess in 31 simulations, where covariance step failed

### Distribution of confidence intervals

**Fig. 2:**
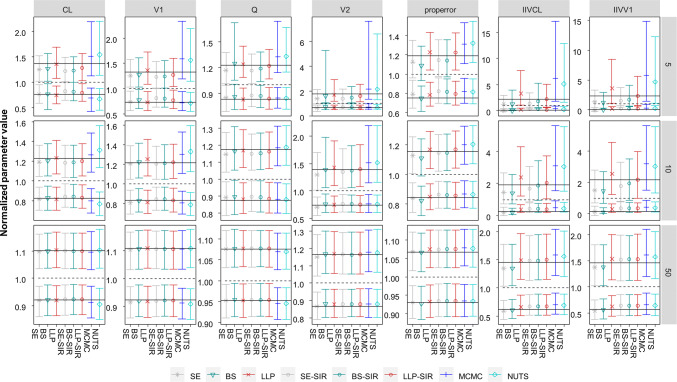
Normalized 10th and 90th percentile of the upper and lower limit of the 95% CI of the parameter uncertainty by parameter and evaluation approach across datasets containing 5–50 subjects. Black lines indicate the 95% confidence interval determined by stochastic simulation and estimation; dashed lines indicate the true parameter value

The distribution of the CIs determined by the different methods is presented in Fig. 2. The parameter uncertainty assessed by the different methods was in parts highly variable, in particular in the case of very small datasets (five subjects) for all parameters, and for small datasets (ten subjects) especially for IIV parameters.

For SE, the 80% CI around the upper limit of the 95% CI was overall too low for the IIVs and its lower boundary was even lower than the true value (respectively 1, when normalized) for small and very small datasets (e.g. 0.74 for IIVCL in small datasets containing ten subjects).

For BS, a similar pattern regarding the CIs of the upper limit was observed, which were also too low and crossed 1 for the IIVs in small and very small datasets.

The LLP provided high uncertainty around the upper limit of the CI of the IIVs in small and very small datasets. For example for IIVCL for datasets with five subjects, the 80% CI of the upper limit of the 95% CI was 1.1–7.7 with a median of 3.3 while the upper limit of the SSE reference was 2.2. LLP varied less as compared to all SIRs for the lower limit of the IIV parameters.

For BAY, the highest uncertainty around the upper limits of the IIV parameters and V2 was observed for datasets with ≤ 10 subjects. While small heterogeneity between SE, BS, LLP and SIR in the CIs of structural parameters in datasets containing 10 subjects was found, BAY provided different CIs and was only more in line with the other methods for datasets containing 50 subjects. The uncertainty of the lower limit of the 95% CI was smaller and more in line with other methods.

For SIR, the CIs around the upper percentile were differing depending on their proposal distribution: The SE-SIR and the BS-SIR derived upper CI limits varied less as compared to the LLP-SIR. However, for IIVs in datasets containing ≤ 10 subjects, the 80% CI around the upper limit crossed 1 more markedly for SE-SIR and BS-SIR than for LLP-SIR. In general, the SIR results followed the tendency of their proposal distribution but improved them with regard to the SSE reference. The lower limit of the IIVs was captured well by all SIR methods, but drawing reliant conclusions is problematic, since the lower bound of the CI of the IIVs tended to zero.

### Evaluation of coverage

**Fig. 3:**
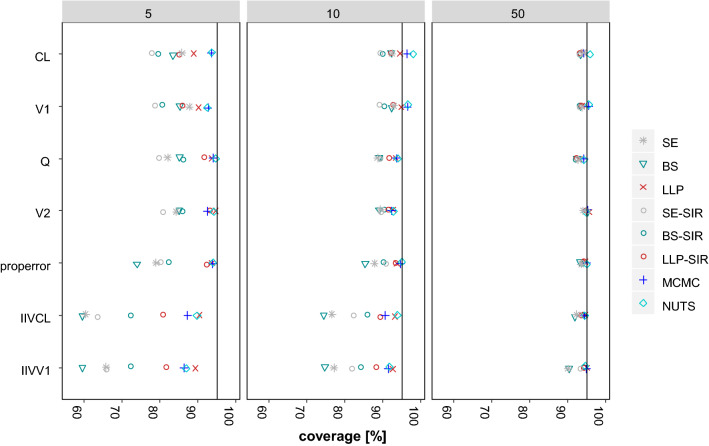
Coverage of the 95% CIs by parameter and evaluation approach across datasets containing 5–50 subjects

The results of the evaluation of the coverage of all included methods is presented in Fig. 3. For a 95% CI, a coverage of the true value of 95% is to be expected in theory. Indeed, for all structural parameters in regular datasets with 50 subjects, the coverage was approximately 95% (92%–95.9% over all methods and all structural parameters). Yet, for the small and very small datasets containing ≤ 10 subjects, the coverage was heterogeneous among the methods (77.7%–94.7% and 88.6%–98.0%, for datasets with five and ten subjects, respectively, over all methods and all structural parameters). In general, the structural parameters were covered better than the variability parameters, but even for CL, the coverage was < 90% for all methods, except BAY (93.5–93.6%) in very small datasets with five subjects.

SE did not cover adequately the true parameters for small and very small datasets. For example, in small datasets containing ten subjects, median coverage (range) was 90.9% (88.6–92.9%) for structural parameters, 76.8% (76.6–77.1%) for IIV parameters.

BS did provide the lowest coverage rates for the IIV parameters in small and very small datasets. Here, in small datasets containing ten subjects, median coverage (range) was 90.6% (89.0–92.3%) for structural parameters, 74.6% (74.4–74.7%) for IIV parameters.

LLP provided the overall highest coverage without exceeding 95% coverage substantially (88.9–95.6% across all parameters and dataset sizes). This was reflected also in small datasets containing ten subjects, where median coverage (range) was 93.9% (92.6–94.8%) for structural parameters and 92.9% (92.6–93.1%) for IIV parameters.

BAY provided highest coverage rates. Overall, coverage by NUTS was closest to 95% indicated by the lowest relative root mean squared error across all dataset sizes and parameters, that was in range with MCMC and LLP (3.3%, 2.7%, 2.8% for MCMC, NUTS and LLP, respectively). BAY exceeded coverage rates of 95% to the highest extend but was varying to a small amount across all dataset sizes and parameters (86.3%–98.0%).

For SIR, the coverages were dependent on the respective proposal distribution. For example, in small datasets containing ten subjects, median coverage (range) by SE-SIR was 89.3% (89.0–89.5%) for structural parameters, 82.1% (81.8–82.3%) for IIV parameters and by BS-SIR was 90.2% (89.3%–90.4%) for structural parameters, 85.0% (84.1%–85.8%) for IIV parameters. The highest coverage amongst the evaluated SIR methods in small and very small datasets was reached by the LLP-SIR. Median coverage (range) for LLP-SIR in small datasets containing 10 subjects was 91.9% (91.5%–92.8%) for structural parameters and 88.8% (88.2%–89.3%) for IIV parameters.

The pattern in the coverages reached by the 40–90% CIs was similar with respect to the different methods, parameters and number of subjects in the dataset as compared to the 95% CI (Supplementary Fig. 2).

### Clinical dataset example

**Fig. 4:**
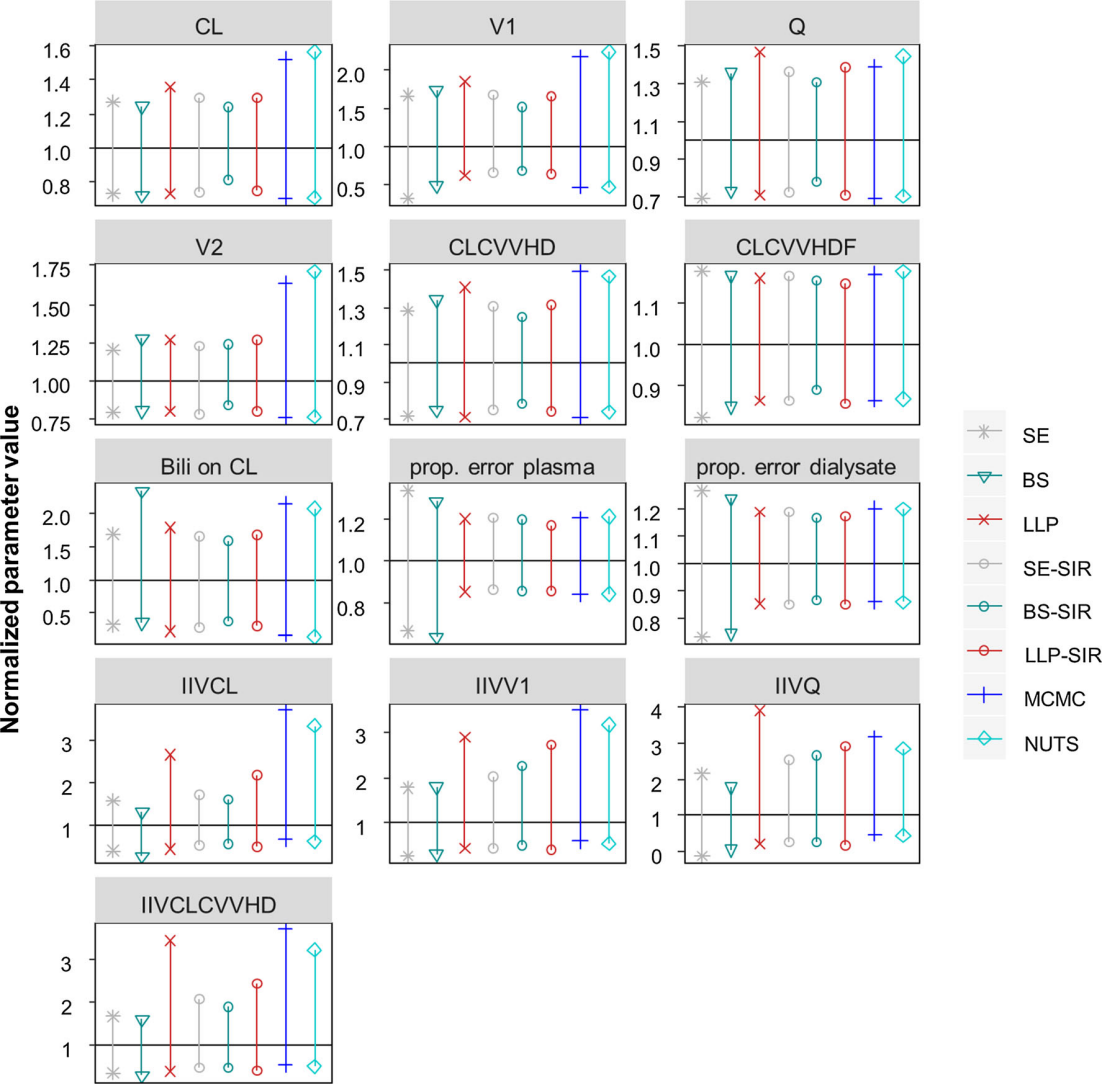
Normalized parameter uncertainty (95% CI) by parameter and evaluation approach in the real data example. CVVHD(F): continuous veno-venous hemodialysis (hemodiafiltration); prop. error plasma (dialysate): proportional error of plasma (dialysate) measurements; Bili: bilirubin

The real dataset example including 11 patients revealed a similar pattern of the evaluated methods compared to the simulation results (Fig. 4). The uncertainty for the structural parameters was similar when determined by SE, BS, LLP, SE-SIR, BS-SIR and LLP-SIR and increased when determined with BAY. However, the uncertainty calculated via SE, the only tested method that did not allow skewness, differed from the others, when a skewed uncertainty was found by BS, LLP, BAY and SIR (e.g. 0.34–1.66 vs. 0.64–1.66 normalized uncertainty for V1 as determined by SE and LLP-SIR, respectively). For IIV parameters, the predicted parameter uncertainty was higher and varied more substantially. LLP and BAY provided the largest 95% CI for IIVs, while SE and BS gave much smaller CIs. The SIR results were dependent on the proposal distribution and the LLP-SIR suggested higher uncertainty than SE-SIR and BS-SIR for all IIV parameters.

## Discussion

The present study comprises an extensive comparison of the performance of different techniques to assess parameter uncertainty in small datasets. The parameter uncertainty determined by SE, BS, LLP, BAY, SE-SIR, BS-SIR and LLP-SIR differed especially for small and very small datasets with ≤ 10 subjects, while the differences were less marked in larger datasets with 50 subjects. The highest influence was seen on parameters describing the random effects, while fixed effect parameters of the non-linear mixed effect models were less affected. Both, low number of subjects in dataset size and random parameter effects were factors increasing uncertainty. Accordingly, the higher the parameter uncertainty was, the higher were the differences in the performance of the methods to assess parameter uncertainty (Supplementary Figure S3). For a parameter uncertainty < 15% estimated by SE, all methods provided similar results regarding the alignment with the SSE reference. Despite this homogeneity for estimated parameter uncertainty at < 15% by SE, the coverage was highly variable and even below 80% for some methods (Supplementary Figure S4). The different behavior of the methods to determine parameter uncertainty was confirmed in the clinical dataset example.

SE and BS were not in line with the SSE results for datasets with ≤ 10 subjects. Especially for IIV parameters, the estimated CIs were too narrow and the IIV was underestimated in median. In very small datasets with 5 subjects also the CIs of structural PK parameters were estimated too narrow. Moreover, the coverage rates of SE and BS were alarmingly low. Even for regular datasets with 50 subjects, the parameter estimates of the IIV were better covered by LLP, BAY and SIR than SE and BS (Fig. 3). While it is widely accepted that the SE is less robust and unreliable, BS is still considered gold standard to assess parameter uncertainty. However, our results corroborate the notion, that both BS and SE are inappropriate methods to assess parameter uncertainty in small datasets [10, 11].

LLP displayed a good performance to derive CIs for the structural parameters over all dataset sizes and was in line with the SSE reference. For the IIV parameters, LLP provided very conservative, yet inflated CIs. In particular, the upper limit of the 95% CI was overestimated in median with a wide distribution across the 1000 simulations for IIV parameters as indicated by the wide distribution of the upper CI limit. The wider CIs might have been beneficial to reach a high coverage rate close to 95% for the methods LLP and BAY. Nonetheless, the coverage was closest to 95% with the LLP and was satisfactory even for small and very small datasets with ≤ 10 subjects.

Standard deviations and point estimates like the mean of the posterior distribution of BAY can provide comparable results to FOCE methods, which was confirmed for datasets containing 50 subjects in this study. BAY is considered a useful method to assess parameter uncertainty for clinical trial simulations [6] and tools are available to provide uncertainty information for simulations [18]. However, for small datasets with ≤ 10 subjects the BAY methods were in median not in line with the FOCE-I based SSE reference and provided inflated confidence intervals that resulted in coverage rates partly exceeding 95%. Overall, the coverage rates reached with NUTS were closest to the desired 95% across all methods and parameters and comparable to coverages reached by LLP and MCMC. The point estimates derived by the MCMC-SSE and NUTS-SSE were overestimating the true variability in these small datasets. A potential explanation is the increased influence of outliers, since the MCMC-SSE and NUTS-SSE were based on the mean and not on median of the posterior distribution. Nonetheless, using a different estimation method can lead to a different point estimate and thus can also alter the ‘true’ parameter uncertainty. Consequently, the estimated parameter uncertainty by BAY was more in line with BAY-based SSE runs, i.e. the MCMC-SSE and NUTS-SSE. A systematic comparison between estimation methods and their respective parameter uncertainty was beyond the scope of the presented study, which focused on typical frequentist FOCE-I-based estimation. Yet, the obtained results in this study are in line with Dartois et al. [10], who observed the same tendency of larger parameter uncertainty estimated by BAY.

The SIR represents a recent addition to the pharmacometric community to assess parameter uncertainty [9]. A SIR run requires a proposal distribution. Usually, the variance covariance matrix is used. Alternatively, a small BS run has been also proposed in case the covariance step failed [9]. However, it is unclear which proposal distribution might provide optimal results leading to accurate CIs with high coverage. Our study showed that the CIs derived by the SIR method were dependent on the respective proposal distribution, which was particularly important in small datasets with ≤ 10 subjects. Contrarily, for datasets with 50 subjects, all studied proposal distributions were appropriate and the SIR results were virtually identical when comparing their central tendency and coverage rates.

The LLP-SIR, i.e. the SIR using an LLP run as proposal, was superior as compared to the SE-SIR and BS-SIR with regard to the alignment with the SSE reference (Fig. 1) and the coverage of the true parameter (Fig. 3). An explanation for this behavior of the SIR technique can be found in the way the algorithm operates: If the proposal distribution is narrower than the true distribution, it is more difficult for the SIR algorithm to flatten the distribution than to narrow it. Therefore, an improper proposal distribution requires diagnosis and inflated proposal distributions might be necessary [9]. Graphical evaluation is recommended to detect limitations in the proposal distribution, the number of sample vectors or the ratio of sample vectors to resample vectors. While other proposal distributions could provide valuable information to the SIR method regarding the covariances (non-diagonal elements), this was not possible with the LLP method. The LLP was a natural candidate for the investigation of benefits in combining methods to derive parameter uncertainty for small datasets with SIR due to missing covariance handling and the option of providing only diagonal elements as input to the SIR-PsN routine. However, in scenarios with highly correlated parameters other methods like importance-sampling variance covariance matrix (IMP) in combination with the SIR could be of value, in case SIR alone could not overcome this missing information. Our study revealed that the LLP might provide a useful proposal distribution to SIR. Combining LLP and SIR helps to also overcome drawbacks of the LLP technique itself, i.e. inflated CIs and univariate parameter uncertainty. The LLP-SIR was successfully applied to a clinical small-n dataset example and tendencies observed here were in line with the simulation results. Accordingly, we cannot identify barriers to overcome the usage of BS or SE in practice for small datasets by using BAY, LLP or LLP-SIR instead.

Some limitations of this study shall be discussed. The in-silico study was performed in NONMEM using FOCE-I in a single case of a two-compartment model. Although the scenario is typical for a small-n study, the results might be different in substantially different scenarios. In contrast to BAY estimation methods, where parameter uncertainty via the posterior distribution is available, for FOCE-I the best way to assess parameter uncertainty is unclear and was therefore in the focus of this study. Not all existing approaches to assess parameter uncertainty were compared, such as parametric BS or N-dimensional LLP, but these approaches are less common or not used, respectively, in the pharmacometric community.

To conclude, the approach used to assess parameter uncertainty requires thorough consideration and not every method, which is appropriate and well accepted for regular and large datasets, is suitable for small datasets. LLP and BAY provided the best coverages regarding datasets containing ≤ 10 subjects while LLP was not over exceeding the desired coverage rates. LLP and in particular BAY provided conservative confidence intervals. An appropriate coverage close to the anticipated coverage is essential for interpretation of the CIs. Hence, LLP and BAY might be preferred techniques when this information is in the focus. SIR was sensitive to the proposal distribution and benefited from LLP as a proposal distribution as compared to SE or BS. The LLP-SIR provided the highest agreement of the parameter uncertainty distribution with the SSE reference. Hence, the LLP-SIR might be the preferred technique when parameter uncertainty is included in stochastic simulations.

## Electronic supplementary material

Below is the link to the electronic supplementary material.

Supplementary file1 (DOCX 1408 kb)
