# Supplementary material for: Assessing parameter uncertainty in small-n pharmacometric analyses: value of the log-likelihood profiling-based sampling importance resampling (LLP-SIR) technique
Source: J Pharmacokinet Pharmacodyn. 2020 Apr 4;47(3):219–28. doi: 10.1007/s10928-020-09682-4 (PMC7289778; doi:10.1007/s10928-020-09682-4)
Supplement: Supplementary file 1 — Supplementary file1 (DOCX 1408 kb) [file 10928_2020_9682_MOESM1_ESM.docx]

**Supplement Table 1** Number of parameters over 1000 simulations where the relative standard error (rse) derived by LLP for LLP-SIR was >=200% and accordingly set to 200%.

| **Parameter** | **Number of rse >=200% (n=1000 simulations)** | | |
| --- | --- | --- | --- |
| **Subjects in dataset** | **5** | **10** | **50** |
| All parameters (n=7) | 166 | 4 | 0 |
| CL | 1 | 0 | 0 |
| V2 | 16 | 0 | 0 |
| IIVCL | 23 | 0 | 0 |
| IIVV1 | 126 | 4 | 0 |


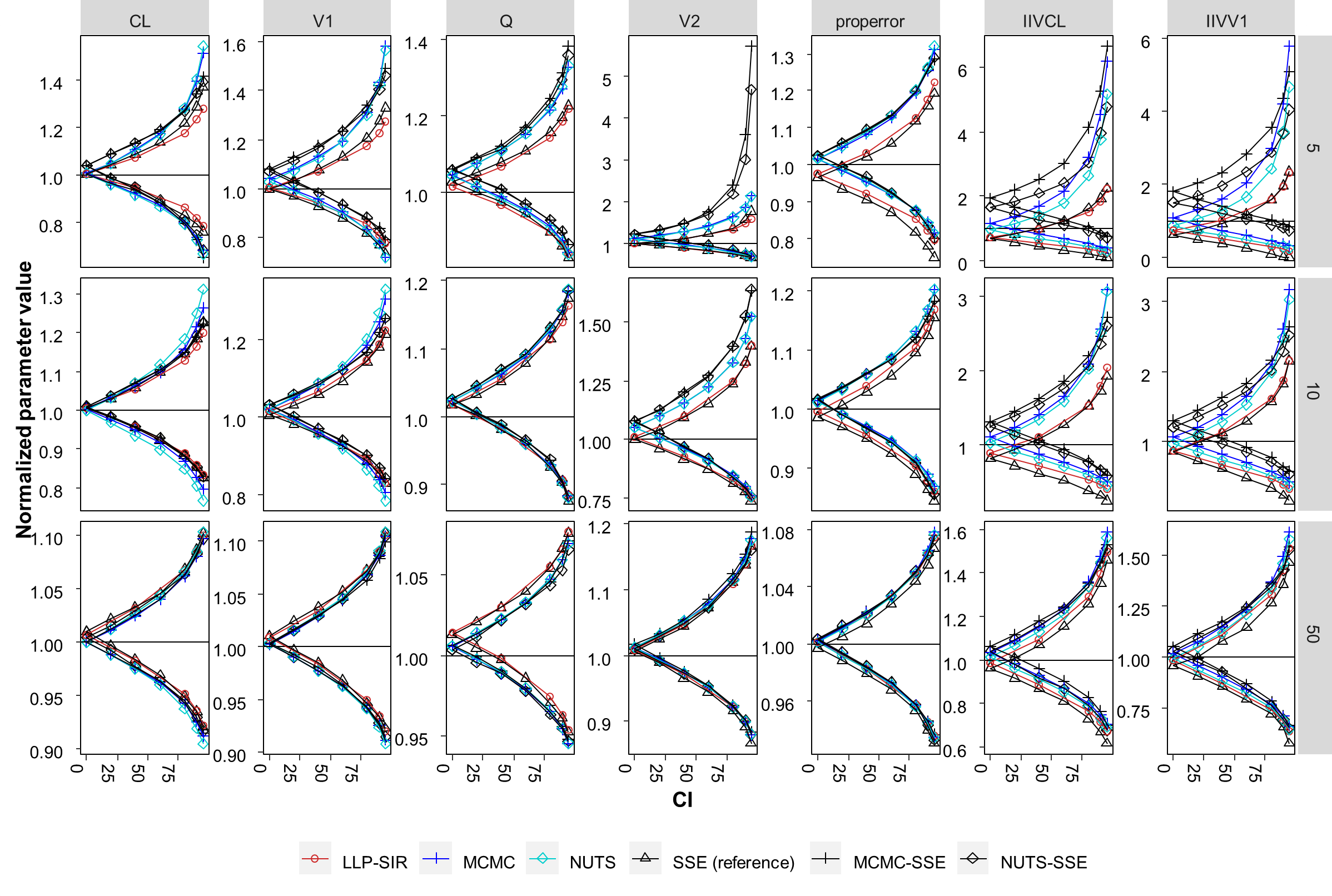


**Fig S1** Normalized median of the parameter uncertainty expressed as 0–95% confidence intervals (CI) by parameter and evaluation approach across datasets containing 5–50 subjects compared to FOCE-I based stochastic simulation and estimation (SSE) ‘reference’ CIs, Markov Chain Monte Carlo Bayesian analysis (MCMC) SSE and no-u-turn sampling (NUTS) MCMC SSE. CIs calculated from the LLP-SIR: sampling importance resampling on LLP based proposal distribution, MCMC, NUTS. IIV: interindividual variability. N=1000 simulations


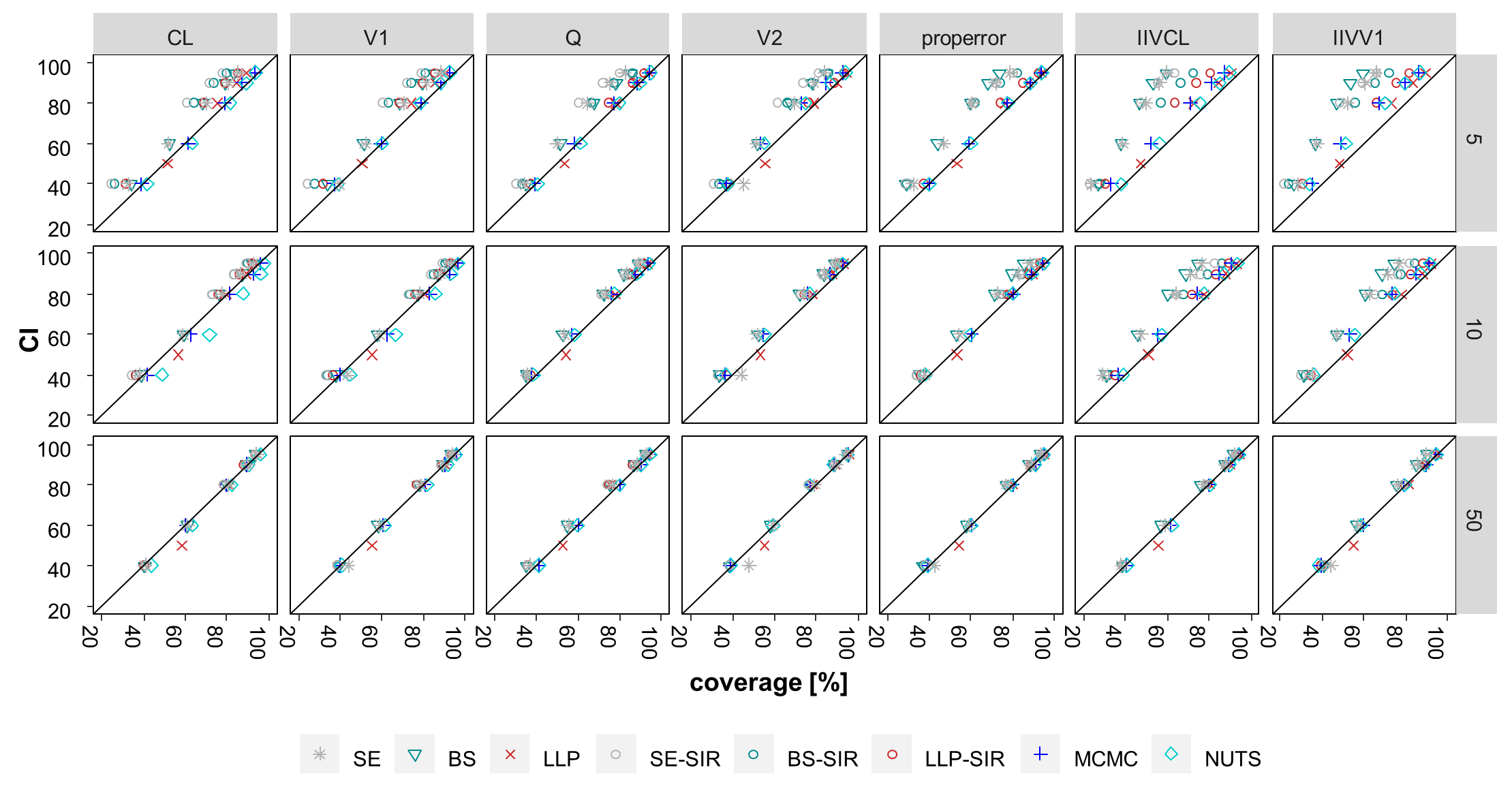


**Fig S2** Coverage of the 40%–95% CIs by parameter and evaluation approach across datasets containing 5–50 subjects


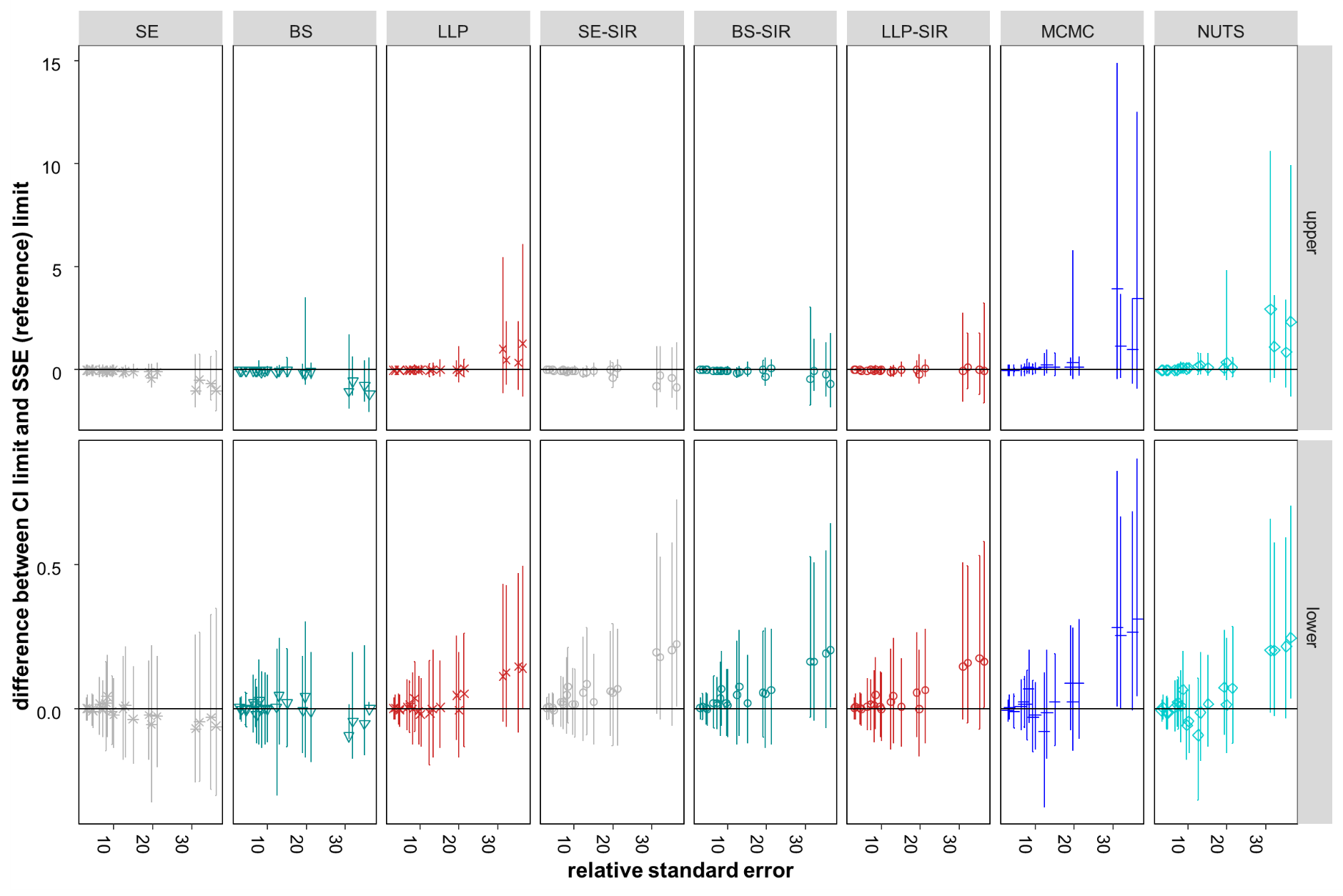


**Fig S3** Difference between the median (point), 10^th^ and 90^th^ percentile (error bar) of the 95% CI of the parameter uncertainty derived by the different methods and the SSE (reference) limits by lower and upper boundary against the parameter uncertainty derived by SE in median for the parameter by number of subjects in the dataset. Note the different scaling of the y-axis for the upper and lower limit.


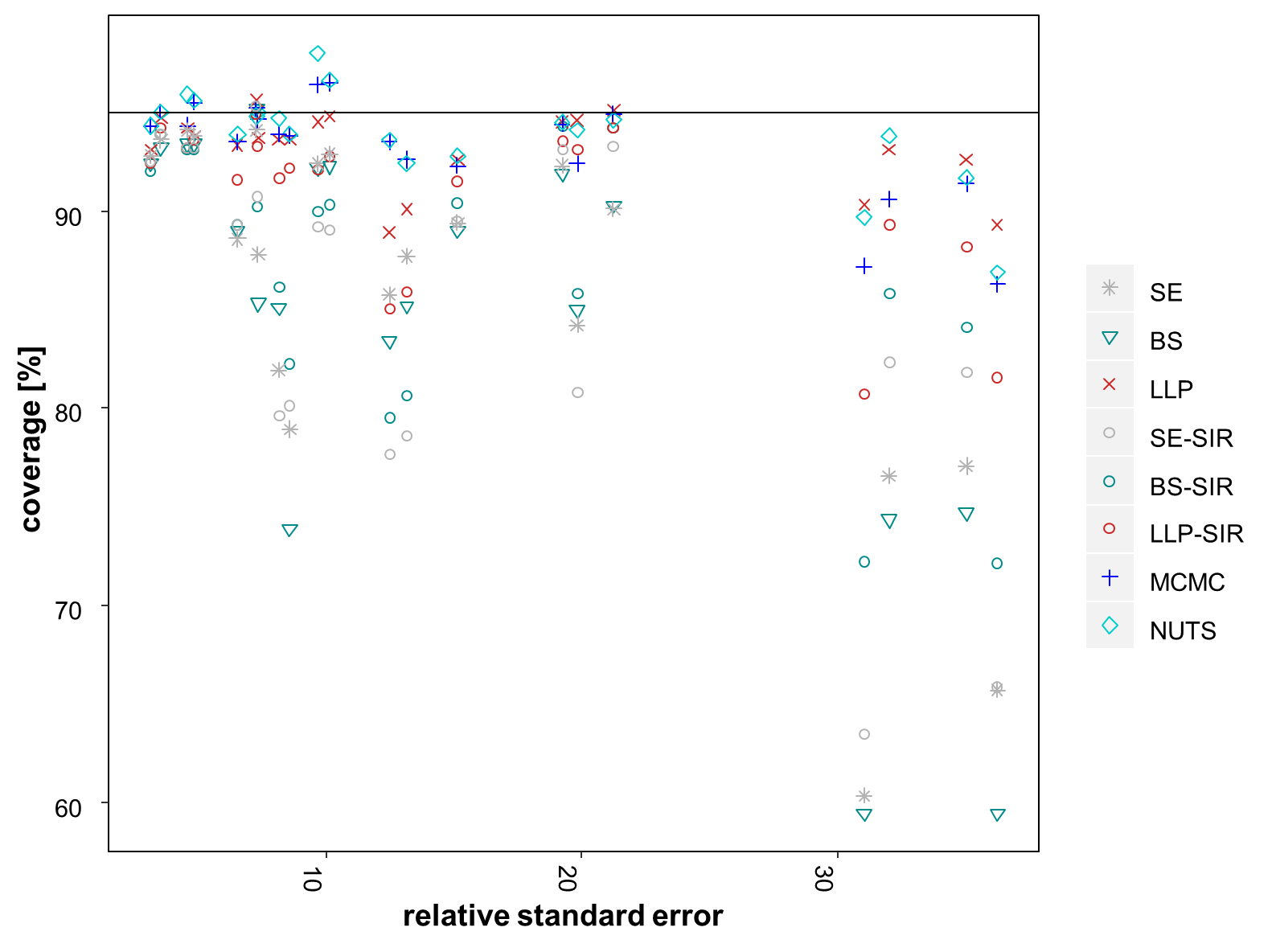


**Fig S4** Coverage of the 95% CIs against the parameter uncertainty derived by SE in median for the parameter by method and number of subjects in the dataset.
